# Injections frequency and health care costs in patients treated with aflibercept compared to ranibizumab: new real-life evidence from Switzerland

**DOI:** 10.1186/s12886-017-0617-x

**Published:** 2017-12-04

**Authors:** Oliver Reich, Martin K. Schmid, Roland Rapold, Lucas M. Bachmann, Eva Blozik

**Affiliations:** 1 Department of Health Sciences, Helsana Group, P.O. Box, Zürich, Switzerland; 2 Eye Clinic, Cantonal Hospital of Lucerne, Lucerne, Switzerland; 3 medignition Inc. Research Consultants, Zürich, Switzerland; 4 Institute of General Practice, University Medical Centre Hamburg-Eppendorf, Hamburg, Germany; 5 Division of General Practice, University Medical Centre Freiburg, Freiburg, Germany

**Keywords:** Claims data, Health insurance, Cost analysis, Macular degeneration, Aflibercept, Ranibizumab

## Abstract

**Background:**

Previous analyses of real-life data indicated that injection frequency and health care costs did not differ for anti-VEGF treatment with aflibercept and ranibizumab. The objective of this study was to investigate whether this finding persisted when analysing a longer time period after licensing.

**Methods:**

Retrospective analysis of health insurance claims data of two large Swiss basic health insurance plans including 28% of the Swiss population. Patients qualified for inclusion if aflibercept or ranibizumab treatment had been initiated between June 1, 2013 and November 1, 2014. Within this set, patients with at least 12 months of continuous insurance enrolment in the previous year, 12-month follow-up, and without change of anti-VEGF drug were considered. We examined the distribution of demographic data and patient characteristics between those receiving ranibizumab and those receiving aflibercept. Numbers of injections and associated health care expenditures observed during the 12-month follow-up period after incident treatment were the two outcomes considered. In multivariate regression analyses, controlling for possible confounding factors, we compared differences in these two outcomes between patients treated with aflibercept as compared to ranibizumab.

**Results:**

A total of 3′058 patients were analysed, 790 (26%) receiving aflibercept and 2`268 receiving ranibizumab (74%). The use of aflibercept (average number of injections 6.2) as compared to ranibizumab (average number of injections 5.7) in the follow-up period of 1 to 12 months, was associated with a 12% increase in the injection frequency (95% confidence interval (CI) 6–17%; *p* < 0.001).

**Conclusions:**

Real-life data contradicts the assumption that aflibercept is used less frequently as compared to ranibizumab. This results in similar total health care expenditures for both anti-VEGF agents.

## Background

The anti-vascular endothelial growth factor (anti-VEGF) medications, ranibizumab (Lucentis®) and aflibercept (Eylea®), were licensed during the evaluation period to treat wet age-related macular degeneration (AMD), and retinal vein occlusion (only central vein occlusions (CVO) for aflibercept), and ranibizumab was also licensed for the treatment of diabetic macular edema (DME) [1, 2].

In contrast to ranibizumab, which only binds to VEGF-A [2–4], aflibercept also binds to VEGF-B and placental growth factor, two additional factors associated with neovascularization [5]. A mathematical model revealing stronger binding affinity of aflibercept to VEGF-165 when compared to ranibizumab suggested that treatment intervals can be extended due to the longer duration of action [6]. The label recommends administration of ranibizumab once monthly until stabilisation or inactivity of the disease is achieved, followed by an individualized treatment interval “as needed”. For aflibercept, the Swiss label recommends to administer the product once a month for the first 3 months followed by once every 2 months [1]. However, the two products are currently considered as equivalent alternatives for the treatment of AMD and CVO [7].

In principle, intravitreal injections should be done as often as necessary and as seldom as possible to reduce the burden of treatment and risks such as endophthalmitis and atrophy [8]. If one of the two drugs would show a clear advantage over the other in terms of risk and treatment burden recommendations and clinical practice guidelines should be adapted accordingly. Currently, two alternative treatment strategies are gaining popularity among retina specialists [9]. As opposed to fix monthly or bimonthly injection schemes, Pro re nata (PRN) means that injections are carried out as needed. The Treat & Extend (T&E) protocol is based on the response to the last injection and decision to reduce or prolong injection intervals is being taken accordingly [7, 9, 10].

However, a recent analysis showed that – opposed to an assumed decrease in injection frequency – injection frequencies in real life increased significantly from 2012 to 2014 [11]. Corroborating these findings, a previous study assessing the delivery of anti-VEGF treatment in Switzerland revealed that aflibercept and ranibizumab were used in a similar fashion, resulting in similar total health care expenditures [1]. However, the Swiss study was done very shortly after licensing of aflibercept, and the follow-up of 6 months might have been too short to show differences that should be observed after the 3-month loading phase.

Therefore, this study investigated, whether the injection pattern of previous real-life studies persisted when investigating a time interval of 12 months starting 6 months after approval of aflibercept, and whether the two drugs differed in terms of health care expenditures.

## Methods

### Study population and context of the study

Health insurance is mandatory in Switzerland, and the basic benefit package is the same in the entire country. The present study included health insurance claims of adult persons residing in Switzerland from two basic health insurances, the Helsana Group (approximately 1.2 million enrolees) and the CSS Group (approximately 1.3 million enrolees). All people residing in Switzerland are required to purchase basic health insurance on a private market of health insurance, which is regulated by federal bodies. The basic health insurance package includes all outpatient or hospital medical treatments deemed appropriate, medically effective, and cost-effective. Supplementary hospital insurance in Switzerland is purchased, if individuals wish further comfort of a semiprivate or private ward or treatment in another canton for personal reasons. Currently, there are 57 insurance companies providing basic health coverage in Switzerland., They offer a range of different premiums and types of health plans from which Swiss residents are free to choose among them managed care health plans [12].

### Identification of patients with macular degeneration

Health insurance claims data in Switzerland do not include diagnoses from the outpatient sector. We therefore used the WHO Anatomical therapeutic chemical (ATC) classification system for drugs to identify patients with MD [13]. All prescription drug items are coded and assigned to a specific pharmaceutical code in our database. On this basis, evidence of intravitreal injections with aflibercept or ranibizumab was acquired by using the corresponding pharmaceutical code. Of this set, patients with at least 12 months of continuous insurance enrolment in the previous year were considered. We included all patients with incident anti-VEGF treatment between 1.6.2013 and 1.11.2014. In order to focus our analysis on patients who had initiated first-line intravitreal anti-VEGF treatment, we excluded individuals who received any ranibizumab or aflibercept treatment in the year before their individual incident treatment date. We also excluded patients in whom both eyes were treated (i.e. less than two prescriptions in the same day) or who received both aflibercept and ranibizumab in the investigated time period. For the purposes of this analysis, we focused on patients with a follow-up period of at least 12 months and anti-VEGF prescriptions from outpatient care only [1].

### Sociodemographic variables, morbidity, costs

Available population characteristics included sex, age, area of residence (Mittelland Region, Northwestern Switzerland, Eastern Switzerland, Ticino, Central Switzerland, Zurich Region, Lake Geneva region) and type of insurance coverage (managed care model, and availability of supplementary private hospital insurance), and presence of any kind of chronic condition using pharmaceutical cost groups. (PCG) If medical diagnosis information is missing in the available data set, PCGs are established individual markers for selected chronic conditions [14]. Our data set also included information on health service use, number of anti-VEGF injections, prescription of drugs other than the aforementioned anti-VEGF drugs, and costs from outpatient and inpatient health care settings. Since the recorded insurance claims cover almost all health care invoices, the data achieve a high degree of completeness. Annual total direct medical costs were obtained from providers’ claims and defined as the total payments made by the mandatory health insurance for outpatient and inpatient services per patient and year. Costs from the outpatient setting comprised payments for office-based physician visits, hospital outpatient visits, paramedical visits, medications, laboratory tests and medical devices. Inpatient costs covered payments for hospital treatments, rehabilitation, nursing home, and emergency transport services. Inpatient costs also cover the cost of medications, laboratory and medical services during the inpatient episode. Costs are given in Swiss Francs (CHF). Only costs related to illness (as opposed to maternity and accident) were considered for this analysis.

### Statistical analysis

Continuous variables were described as the mean or median. Dichotomous variables were described with percentages. In univariate analyses, we examined the distribution of demographic data and patient characteristics between those receiving ranibizumab and those receiving aflibercept. Differences between groups were statistically tested with parametric (chi squared) or nonparametric (Wilcoxon) tests as appropriate. The two outcomes considered were numbers of injections and associated health care expenditures observed during the 12-month follow-up period after incident treatment. We used multivariate regression analysis, controlling for possible confounding factors (sex, age, area of residence, franchise), and the previous year utilization measures hospitalization, medication use (as measured using number of different ATC), total health care cost and presence of PCG to compare differences in the number of injections and costs between patients receiving ranibizumab and those receiving aflibercept. For sensitivity analyses, we did stratified analyses for patients who received both ranibizumab and aflibercept in the observed period of time (changers) as compared to patients who received only one of both (non-changers). A *p*-value of less than 5 % was considered statistically significant. Analyses were performed using the statistical package R, version 3.2.0. (R Foundation for Statistical Computing, Vienna, Austria).

### Ethical approval

The analysis complied with the Swiss Federal Law on data protection. All data were retrospective, pre-existing, de-identified and anonymized prior to the performed analysis. As this study is not subject to the Swiss Federal Law on human research (Humanforschungsgesetz), an ethical approval and consent of patients was not needed.

## Results

The analyses included a total of 3′058 patients of which 790 (26%) received aflibercept and 2`268 received ranibizumab (74%). Patients with aflibercept were about 3 years older and more likely to be female and to be enrolled in a managed care health plan than patients receiving ranibizumab. However, patients with ranibizumab had higher health care expenditures in the preceding year. They also used health services more frequently (Table 1).

**Table 1:** Socioeconomic, clinical and health utilization characteristics of the study population stratified for treatment with ranibizumab or aflibercept

Characteristic	Maeasure	Aflibercept	Ranibizumab	Total	p
Sample size	N (%)	790 (25.8)	2′268 (74.2)	3′058 (100)	
Age	Mean (Median)	79.3 (81)	75.9 (78)	76.7 (79)	< 0.001
19–25 years	N (%)	0 (0.0)	4 (0.2)	4 (0.1)	< 0.001
26–30 years	N (%)	0 (0.0)	2 (0.1)	2 (0.1)	
31–35 years	N (%)	0 (0.0)	8 (0.4)	8 (0.3)	
36–40 years	N (%)	1 (0.1)	11 (0.5)	12 (0.4)	
41–45 years	N (%)	3 (0.4)	30 (1.3)	33 (1.1)	
46–50 years	N (%)	0 (0.0)	34 (1.5)	34 (1.1)	
51–55 years	N (%)	8 (1.0)	52 (2.3)	60 (2.0)	
56–60 years ‘	N (%)	10 (1.3)	102 (4.5)	112 (3.7)	
61–65 years ‘	N (%)	29 (3.7)	136 (6.0)	165 (5.4)	
66–70 years ‘	N (%)	74 (9.4)	245 (10.8)	319 (10.4)	
71–75 years ‘	N (%)	96 (12.2)	313 (13.8)	409 (13.4)	
76–80 years	N (%)	170 (21.5)	395 (17.4)	565 (18.5)	
81–85 years	N (%)	198 (25.1)	481 (21.2)	679 (22.2)	
86–90 years	N (%)	145 (18.4)	321 (14.2)	466 (15.2)	
≥ 91 years	N (%)	56 (7.1)	134 (5.9)	190 (6.2)	
Female	N (%)	521 (65.9)	1′315 (58)	1′836 (60)	< 0.001
Managed Care health plan	N (%)	319 (40.4)	800 (35.3)	1′119 (36.6)	0.012
Suppl. general inpat. Insurance	N (%)	379 (48)	1′097 (48.4)	1′476 (48.3)	0.881
Total cost (CHF)^a^	Mean (Median)	9′422 (5′752)	11′461 (6′943)	10′935 (6′564)	< 0.001
Outpatient cost (CHF)^a^	Mean (Median)	7′536 (5′280)	8′870 (6′134)	8′525 (5′932)	< 0.001
Medication cost (CHF)^a^	Mean (Median)	1′898 (1′280)	2′338 (1′481)	2′224 (1′412)	< 0.001
Inpatient cost (CHF)^a^	Mean (Median)	2′279 (0)	3′151 (0)	2′926 (0)	0.006
Nr. of hospitalisations^a^	Mean (Median)	0.3 (0)	0.4 (0)	0.4 (0)	0.016
Nr. of outpatient visits^a^	Mean (Median)	15.4 (13)	15.8 (13)	15.7 (13)	0.159
Nr. of hosp. Outp. visits^a^	Mean (Median)	3.9 (2)	5.3 (2)	5 (2)	< 0.001
Nr. of PCGs^a^	Mean (Median)	2.3 (2)	2.4 (2)	2.3 (2)	0.262
Nr. of different ATC^a^	Mean (Median)	14.5 (13)	15.4 (14)	15.2 (14)	0.006

*CHF* Swiss Francs, *PCG* Pharmacy Cost Group, *ATC* anatomical therapeutic chemical classification system

^a^assessed in the preceding year

Table 2 displays the number of injections in the year following incident treatment. When comparing the period following the loading phase of 3 months, the injection frequency of aflibercept and ranibizumab did not differ. When investigating the complete year following incident treatment, aflibercept was used slightly, but statistically significantly more frequently.

**Table 2:** Frequency of injection in the year following incident treatment of aflibercept and ranibizumab

Number of injections	1	2	3	4	5–6	7–9	≥ 10	Mean (median	p
Months 1–12 after incident treatment									
Total (*N* = 3′058)	250 (8.2)	235 (7.7)	558 (18.2)	312 (10.2)	582 (19.0)	688 (22.5)	433 (14.2)	5.8 (5)	
Aflibercept (*N* = 790)	52 (6.6)	39 (4.9)	141 (17.8)	67 (8.5)	151 (19.1)	227 (28.7)	113 (14.3)	6.2 (6)	
Ranibizumab (*N* = 2′268)	198 (8.7)	196 (8.6)	417 (18.4)	245 (10.8)	431 (19.0)	461 (20.3)	320 (14.1)	5.7 (5)	< 0.001
Months 4–12 after incident treatment									
Aflibercept (*N* = 589)	73 (12.4)	79 (13.4)	94 (16.0)	95 (16.1)	138 (23.4)	82 (13.9)	28 (4.8)	4.4 (4)	
Ranibizumab (*N* = 1′499)	260 (17.3	212 (14.1	248 (16.5)	173 (11.5)	318 (21.2)	209 (13.9)	79 (5.3)	4.3 (4)	
Total (*N* = 2′088)	333 (15.9)	291 (13.9)	342 (16.4)	268 (12.8)	456 (21.8)	291 (13.9)	107 (5.1)	4.3 (4)	0.066

**Fig. 1:**
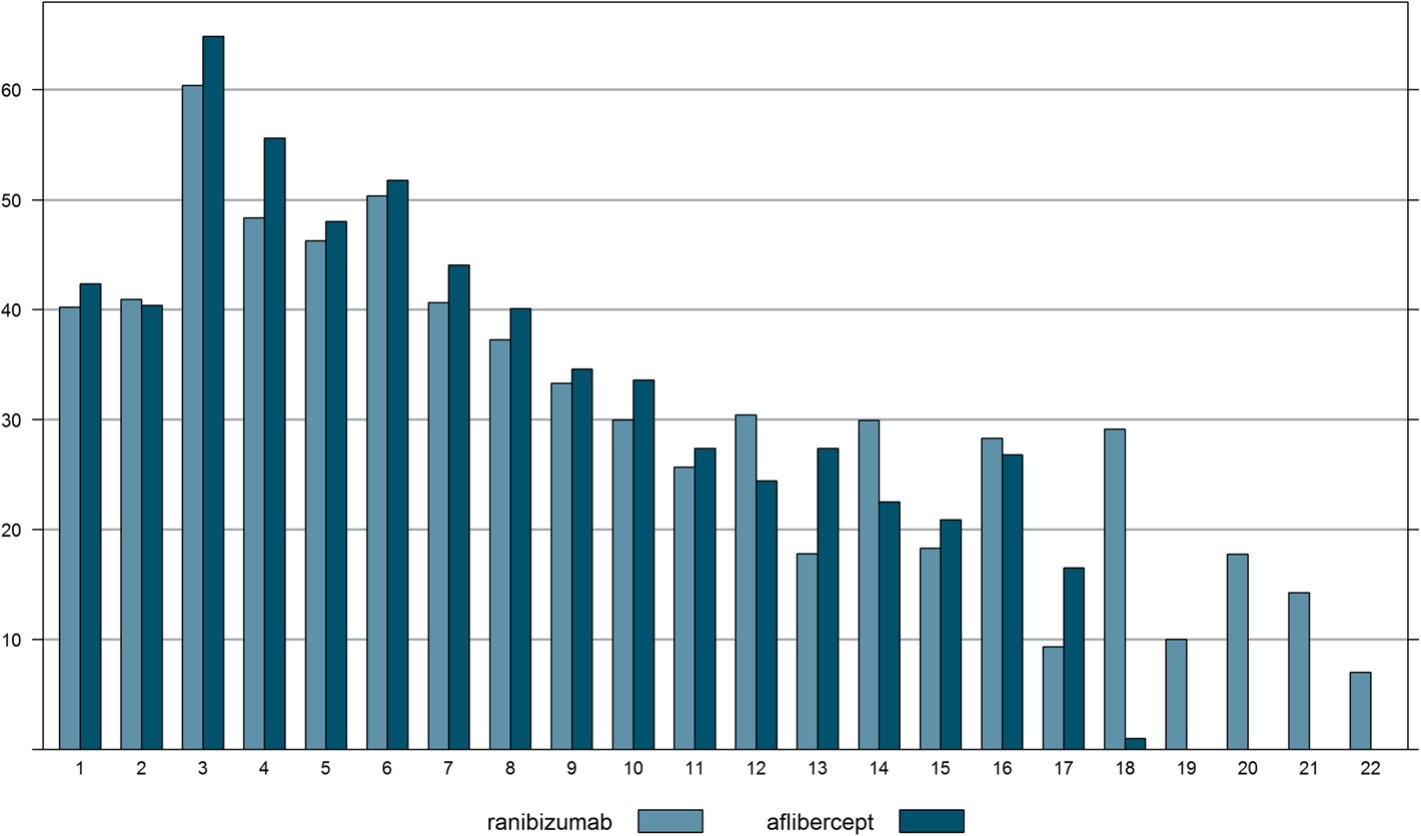
Mean time interval in days between injections of aflibercept and ranibizumab in the months 1–12 following incident treatment. *Light blue*: ranibizumab; *dark blue*: aflibercept

Mean time intervals between injections of ranibizumab ranged from 33 to 60 days with the median fluctuating around approximately 30 days. Aflibercept was injected in mean intervals of 35 to 65 days with the median ranging from 30 to 49. The differences were statistically significant for 5 out of 9 intervals, but the absolute difference in days ranged from 0.5 to 7.2 days for the mean and from 0 to 11 for the median (Table 3). For the intervals following the loading phase (4–9) the difference was considerably shorter than expected, given the recommendation of bimonthly injections of aflibercept. Figure 1 illustrates how injection intervals changed during the course of treatment for the two medications. In the initial phase, treatment with ranibizumab and aflibercept followed a similar pattern of monthly injections. After that, injection intervals of both medications were prolonged without showing a clear pattern that intervals of aflibercept were twice as long as those of ranibizumab.

**Table 3:** Mean time interval in days between injections of aflibercept and ranibizumab in the months 1–12 following incident treatment

Intervall	Ranibizumab (mean)	Ranibizumab (median)	Ranibizumab (N)	Aflibercept (mean)	Aflibercept (median)	Aflibercept (N)	p-Wert
1	40.2	28	2051	42.3	30	737	0.054
2	40.9	29	1847	40.4	30	694	0.104
3	60.4	42	1419	64.8	49	549	< 0.001
4	48.4	35	1167	55.6	46	478	< 0.001
5	46.3	35	971	48.0	42	417	0.002
6	50.4	42	733	51.8	42	321	0.088
7	40.6	35	564	44.1	42	234	0.006
8	37.3	35	415	40.1	42	157	0.001
9	33.3	31	265	34.6	33.5	92	0.413

Multivariate regression analysis to investigate the association between number of injections and the medication used adjusting for sociodemographic and clinical differences in the underlying population revealed that use of aflibercept as compared to ranibizumab was associated with a 12% increase in the injection frequency (regression coefficient 1.12, 95% confidence interval 1.06–1.17). The estimate was statistically significantly different from zero. With respect to total health care costs, use of aflibercept was associated with a statistically non-significant 4% increase. Apart from socio-demographic characteristics and proxies for health status, both outcomes were significantly influenced by region of residence (Table 4). Sensitivity analyses using stratified analyses for changers as compared to non-changers did not reveal substantial differences.

**Table 4:** Results of multivariate regression analysis to predict the number of injections of aflibercept compared to ranibizumab and total health care costs in patients receiving aflibercept compared to ranibizumab in the months 1–12 following incident treatment

	Number of injections			Total healthcare costs		
Independent variable	Coefficient	95% Confidence interval	p	Coefficient	95% Confidence interval	p
Use of aflibercept	1.12	(1.06–1.17)	< 0.001	1.04	(0.99–1.09)	0.129
Age 61–65	1.13	(1.00–1.27)	0.048	1.06	(0.96–1.18)	0.256
Age 66–70	1.09	(0.98–1.20)	0.104	1.05	(0.96–1.14)	0.329
Age 71–75	1.03	(0.93–1.13)	0.594	1.06	(0.97–1.15)	0.200
Age 76–80	1.02	(0.93–1.12)	0.616	1.07	(0.99–1.17)	0.088
Age 81–85	1.00	(0.92–1.10)	0.919	1.08	(1.00–1.17)	0.053
Age 86–90	0.94	(0.85–1.03)	0.196	1.05	(0.96–1.14)	0.303
Age 91 +	0.80	(0.71–0.90)	< 0.001	1.03	(0.93–1.14)	0.599
Female	0.99	(0.94–1.03)	0.577	0.95	(0.91–0.99)	0.016
Franchise ≥ 1000 CHF	0.98	(0.90–1.06)	0.625	0.98	(0.91–1.06)	0.647
Hospitalisation^a^	1.05	(0.98–1.12)	0.178	0.93	(0.88–0.99)	0.019
Mittelland Region	1.06	(0.98–1.14)	0.128	0.94	(0.88–1.00)	0.069
Northwestern Switzerland	1.07	(0.99–1.16)	0.092	1.00	(0.94–1.07)	0.945
Eastern Switzerland	1.11	(1.01–1.21)	0.026	0.93	(0.86–1.00)	0.054
Ticino	0.84	(0.76–0.92)	<0.001	0.88	(0.81–0.96)	0.003
Central Switzerland	1.28	(1.17–1.39)	<0.001	1.04	(0.96–1.12)	0.377
Zürich Region	1.16	(1.07–1.25)	<0.001	0.97	(0.91–1.03)	0.337
1–10 different ATC^a^	0.96	(0.78–1.18)	0.685	1.00	(0.83–1.21)	0.975
11–18 different. ATC^a^	0.93	(0.75–1.15)	0.519	1.05	(0.87–1.28)	0.606
≥ 19 different ATC^a^	0.87	(0.70–1.09)	0.221	1.07	(0.88–1.31)	0.483
1–2 PCGs^a^	1.02	(0.96–1.09)	0.497	1.08	(1.02–1.15)	0.009
2–3PCGs^a^	0.96	(0.88–1.04)	0.310	1.08	(1.01–1.17)	0.032
≥ 4 PCGs^a^	0.99	(0.91–1.07)	0.808	1.15	(1.07–1.24)	0.000
Total cost^a^ (00097–03536 CHF)	0.95	(0.51–1.75)	0.860	1.01	(0.57–1.78)	0.970
Total cost^a^ (03548–06831 CHF)	0.98	(0.53–1.81)	0.947	1.13	(0.64–2.00)	0.670
Total cost^a^ (06838–13,308 CHF)	0.97	(0.53–1.80)	0.933	1.26	(0.71–2.23)	0.429
Total cost^a^ (13321–22,085 CHF)	0.94	(0.50–1.74)	0.837	1.53	(0.86–2.71)	0.149
Total cost^a^ (≥ 22,188 CHF)	0.94	(0.51–1.75)	0.853	2.18	(1.23–3.86)	0.008

*CHF* Swiss Francs, *PCG* Pharmacy Cost Group, *ATC* anatomical therapeutic chemical classification system

^a^assessed in the preceding year

## Discussion

This study provides further real-life evidence that use of aflibercept as an alternative to ranibizumab does not reduce injections frequency and costs. The recommended treatment pattern of a bimonthly injection of aflibercept following a 3-months loading phase is obviously not implemented. Generally, treatment choice does not affect clinical decision making, as for both medications the injection frequency decreases after the 3-months loading phase.

The present study is in line with previous research. In 2013, Johnston et al. published the results of a retrospective analysis of first-line anti-VEGF treatment patterns in AMD based on patient prescription drug claims in the USA. They compared numbers of injections and associated health care expenditures between patients receiving ranibizumab or aflibercept for 6 or 12 months and found no differences between the two drugs [15]. Another study from the USA also found that in routine clinical practice, patients received a comparable number of injections in the first year of treatment [16]. Previous studies also investigated differences in clinical outcomes between both drugs and found no difference in favour of either substance [17, 18].

The previous study from Switzerland investigating treatment patterns of anti-VEGF medications showed that clinicians do not follow the recommendation regarding frequency of injections of aflibercept [1]. This study has been criticised for the investigated time interval being too short after licensing of aflibercept for AMD and CVO, and a follow-up of 6 months being too short to show an effect after the 3-month loading phase. The present study addresses this criticism by using a follow-up of 12 months, investigating a time period starting 6 months after approval of aflibercept. It clearly supports previous findings and contradicts predications of longer injection intervals and/or decreased usage and costs when choosing aflibercept rather than ranibizumab.

The main strength of the present study is that it explores treatment patterns and effects in real-life. Effects observed in clinical trials with carefully selected participants and highly standardised clinical procedures bear the risk to fail in daily clinical routine. The present study uses reimbursement data, and it is unlikely that treatments in the investigated patient population have been missed. This is especially important as currently different treatment strategies, such as fixed, PRN or T&E are being used in clinical routine.

Several limitations need to be considered. Firstly, treatment was not allocated randomly, bearing the risk of selection bias. For example, aflibercept might be considered as “salvage” “therapy” in patients with disease patterns known to be resistant to ranibizumab treatment [16]. Secondly, due to lack of clinical data, we were unable to differentiate between the different indications for anti-VGEF treatment, and it might be possible that our results vary according to the underlying ophthalmologic disease. In addition, the present study did not compare clinical outcomes of treatment with aflibercept and ranibizumab. Thirdly, differences between persons enrolled in different Swiss basic health insurance programmes preclude generalisations to the whole country. However, the study included patients from two different health insurances covering 28% of the Swiss population.

The present study has implications for future research. First, the results of the present study should be correlated with patient-relevant clinical outcomes to investigate the magnitude of the treatment effect and potential differences between the two substances. In addition, further studies should explore reasons why ophthalmologists deviated from the treatment scheme propagated in the package insert, whether these deviations base on PRN or T&E, and whether different administration of drugs resulted in different clinical outcomes. Furthermore, future studies need to differentiate between clinical subgroups of patients i.e. patients with AMD, DME, CVO or BVO (branch vein occlusion). Moreover, our study had a maximum follow-up time of 12 months. Future studies should focus on longer time intervals to investigate treatment patterns after the relatively well-studied treatment period of 12 or 24 months.

### Implications for practice

Current evidence regards aflibercept and ranibizumab as equivalent alternatives for treatment in AMD with respect to safety and efficacy [19]. Our findings indicate that there is also financial equality with a tendency for increased costs related to use of aflibercept. In principal, the bimonthly injection regimen of aflibercept would represent reduced treatment burden and risks associated with frequent injections compared to the monthly dosing scheme of ranibizumab. The results of this study will increase transparency related to the assumptions that were made at the time of approval based on the results of clinical studies. They may be help to foster the discussion about clinical, evidence-based criteria related to the choice of anti-VGEF substances, to the decision for continuation or discontinuation, and for treatment and follow-up intervals. Given the fact that the region of patients´ residence significantly influenced both the number of injections and total health care costs in multivariate analyses, national treatment recommendations might help to reduce these differences in patient management across the country.

## Conclusions

This study increases the body of evidence indicating that treatment patterns of aflibercept and ranibizumab do not differ systematically. Our analysis cannot confirm the postulated benefit of less injections and/or longer intervals between aflibercept treatments. The total health care expenditures for aflibercept and ranibizumab proved to be similar.

## Abbreviations

AMD: Age-related macular degeneration
Anti-VEGF: Anti-vascular endothelial growth factor
ATC: Anatomical therapeutic chemical classification system
BVO: Branch vein occlusion
CHF: Swiss Francs
CI: Confidence interval
CVO: Central vein occlusions
DME: Diabetic macular edema
PCG: Pharmacy Cost Group
PRN: Pro re nata
T&E: Treat & Extend
